# Blood Biomarkers of Sensitization and Asthma

**DOI:** 10.3389/fped.2019.00251

**Published:** 2019-06-19

**Authors:** Hans-Joachim Sonntag, Sarah Filippi, Spyros Pipis, Adnan Custovic

**Affiliations:** ^1^Respiratory Division, National Heart and Lung Institute, Imperial College London, London, United Kingdom; ^2^Department of Mathematics, Imperial College London, London, United Kingdom; ^3^Department of Epidemiology and Biostatistics, School of Public Health, Imperial College London, London, United Kingdom; ^4^Medical School, University of Nicosia, Nicosia, Cyprus; ^5^Department of Paediatrics, Aretaeio Hospital, Nicosia, Cyprus

**Keywords:** asthma, biomarkers, data driven analysis, cohorts, IgE, eosinophils, childhood

## Abstract

Biomarkers are essential to determine different phenotypes of childhood asthma, and for the prediction of response to treatments. In young preschool children with asthma, aeroallergen sensitization, and blood eosinophil count of 300/μL or greater may identify those who can benefit from the daily use of inhaled corticosteroids (ICS). We propose that every preschool child who is considered for ICS treatment should have these two features measured as a minimum before a decision is made on the commencement of long-term preventive treatment. In practice, IgE-mediated sensitization should be considered as a quantifiable variable, i.e., we should use the titer of sIgE antibodies or the size of skin prick test response. A number of other blood biomarkers may prove useful (e.g., allergen-specific IgG/IgE antibody ratios amongst sensitized individuals, component-resolved diagnostics which measures sIgE response to a large number of allergenic molecules, assessment of immune responses to viruses, level of serum CC16, etc.), but it remains unclear whether these can be translated into clinically useful tests. Going forward, a more integrated approach which takes into account multiple domains of asthma, from the pattern of symptoms and blood biomarkers to genetic risk and lung function measures, is needed if we are to move toward a stratified approach to asthma management.

## Introduction

Asthma is a heterogeneous condition with a myriad of definitions in the literature (1), and is characterized by chronic airway inflammation which gives rise to a range of respiratory symptoms including wheeze, cough and shortness of breath (1). Allergic (or atopic) sensitization, measured via skin prick tests (SPT) or specific serum immunoglobulin E (sIgE) levels, has been shown to be a strong risk factor associated with asthma in a number of epidemiological studies (2). However, the paradigm of asthma as a purely atopic disease was challenged at the turn of the millennium, with evidence indicating that less than half of asthma cases in some populations can be attributed to atopy, although this proportion appears higher in children (3). The pathophysiology that is assumed to characterize “atopic” or “allergic” asthma (the underlying sensitization to common allergens causing an immune response when the individual is exposed to them, leading to chronic airway inflammation and acute allergen-mediated airway constriction) is only one facet of asthma, and non-allergic factors must be taken into account (4).

After decades of improvements, in the past 10–15 years little progress has been made against key outcomes in asthma such as hospital admissions and mortality rate (5). A recent Lancet commission on asthma offered a view that the reductionist approach to asthma as a single disease, which persists in clinical practice and research studies, is one of the factors preventing advances in the field (1). Instead, asthma should be seen as an aggregate of several distinct subtypes that are characterized by different pathophysiological mechanisms (often referred to as endotypes) (6, 7). However, as the term endotype refers to a specific disease subtype with clearly defined and unique pathophysiological mechanisms, applying this definition to the current state of knowledge in asthma suggests that no “true” asthma endotypes have been described with certainty to date, and endotyping remains a work in progress (8). The recognition that asthma is heterogeneous has led some to argue that the term “asthma” should be abandoned altogether, since it cannot be regarded as a single disease entity (9).

Biomarkers are needed both to determine the subtype of asthma, and for prediction of response to treatments. This review will focus on the importance of blood biomarkers, most notably serum IgE levels and blood eosinophil counts, and will mainly focus on childhood asthma. Longitudinal trajectories of allergic sensitization during childhood and their relevance to asthma-related conditions will be discussed, as well as different types of modeling required to uncover such trajectories. A further blood biomarker of interest is serum periostin, shown in a recent study to be a better predictor of airway eosinophilia than IgE levels or blood eosinophils (10). However, the usefulness of periostin in pediatric asthma has been questioned, as it is also a marker of bone growth in childhood (11).

Moving beyond blood biomarkers, we present the reader with an overview of further asthma domains, from lung function measurements to genetic findings. Put together, all of this knowledge points to a need for the integration of all aspects of this complex condition, combined with methodological development, as the way forward to a complete understanding of the underlying endotypes and the development of mechanism-based stratified treatments (12).

## Hallmarks of Asthma: From Allergic Sensitization to Markers of Inflammation

### Total IgE Levels and IgE Responses to Specific Allergens Give Clues About Asthma

The presence of specific IgE antibodies measured in blood or by SPTs defines allergic sensitization, and is one of the strongest associates of asthma in epidemiological studies (13, 14). After recognizing particular allergic molecules, cross-linking of IgE molecules on effector cells can trigger the release of mediators of inflammation such as histamine, leukotrienes and interleukins, and cause many of the symptoms that are associated with allergic diseases.

Total level of IgE was also shown to be an important predictor of asthma in population-based studies; this association persists after adjusting for specific IgE levels against several core allergens (dust mite, cat, timothy grass, fungi, and plants) (15). This observation has led to the development of anti-IgE monoclonal antibodies such as omalizumab for the treatment of asthma (16). However, it is important to note that although total IgE level is used to select patients for omalizumab treatment, it does not predict the response to this drug (17). While the development of biologics for the management of asthma has been accelerating, there are still many hurdles toward their more widespread use in clinical practice, particularly in pediatric asthma where relevant clinical trials to confirm safety and efficacy are ongoing (18). Biomarkers that would accurately predict treatment response to different biologics would be very useful. For example, focusing on patients with multiple IgE-mediated comorbidities and polysensitization to a wide range of allergens may be of particular interest in the case of omalizumab, as this pattern of sensitization biomarkers may potentially reflect a subtype of asthma with preferential response, facilitating a more targeted treatment (19). Conversely, biologics can also provide crucially important information to facilitate the discovery of endotypes of asthma, as identification of responders to treatment may point out at the pathways causing disease expression in different subgroups.

A clearer picture about the association between sensitization and asthma can emerge when responses to specific allergens are taken into account (20, 21). For instance, a distinct relationship between fungal allergy and severe asthma has been reported (21), leading to the emergence of a new phenotype of “severe asthma with fungal sensitization (SAFS)” (22, 23). A number of studies have pointed to the significance of IgE response to house dust mite allergens in the development of asthma (24, 25), whist in the areas with low mite allergen levels, other allergens such as Alternaria or furry pets dominate (26). Children with early mite sensitization who are exposed to high levels of dust mite allergens in their homes in the first year of life have diminished lung function as early as age 3 years (27), and are at high risk of having asthma at school age (28). However, despite the evidence of the association between mite sensitization and high exposure with asthma, clinical outcomes reported by different studies investigating mite avoidance as a primary prevention strategy are inconsistent and often confusing. For example, in the Isle of Wight study, mite sensitization and asthma were significantly reduced in the intervention group by age 18 years (29), while the Manchester study reported an increase in mite sensitization (30), and some intervention trials reported no observable effect (31). In line with the recommendation for the recruitment of more specific subgroups of patients for drug trials, the same can be applied to prevention trials, and investigating mite avoidance in a better defined group of children at risk may be more appropriate.

We will return to the relevance of specific allergens and the methodological developments that have helped identify the association between different sensitization patterns and asthma in the next section. However, since a large portion of sensitized individuals do not develop asthma (13), the presence of IgE-mediated sensitization *per se* is not a very useful biomarker for asthma diagnosis. It is important to emphasize that in clinical practice, allergic sensitization should be considered and interpreted as a quantifiable rather than dichotomous trait, i.e., we should use the titer of sIgE antibodies or the size of skin test response instead of relying on a simple presence of “sensitization” determined using arbitrary criteria (32). The level of sIgE antibodies to common inhalant allergens has been shown to be a good predictor of the presence and persistence of childhood asthma, as well as reduced lung function (25, 33). Another important point for interpretation of skin tests and specific IgEs is that patients' age and sex should be taken into account when interpreting these tests in the context of asthma, and age- and sex-specific normative data are urgently needed (34). For example, for any given size of skin test response or the titer of sIgE, boys are more likely to have asthma, particularly in pre-school age (34).

Of note, control mechanisms mediated via allergen-specific IgG may also play an important role, and may help differentiate between benign sensitization (i.e., sensitization without symptoms of allergic disease) and pathologic sensitization (i.e., sensitization leading to clinical symptoms) (35). Amongst sensitized individuals, the IgG/IgE antibody ratios for dust mite and grass allergens were found to be lower among children who had asthma and allergic rhinitis, respectively, compared to asymptomatic atopic children in two birth cohorts in the UK and Australia, indicating that allergen-specific IgG antibodies in sensitized individuals may protect against expression of symptoms and the development of asthma (36). Furthermore, among sensitized asthmatics, low house dust mite-specific IgG/IgE antibody ratio was associated with more severe disease (36). Thus, sIgG_1_/sIgE ratio might be a more relevant biomarker of asthma presence and severity compared to sIgE levels alone (36), but these findings are yet to be translated into useful biomarkers for clinical practice. However, it is of note that the onset of IgE sensitization to a particular molecule has been found to almost always coincide with a strong IgG_1_ response to the same molecule (37, 38). It is thus not clear whether IgG_1_ in itself has a protective effect. In the context of allergen-specific immunotherapy, IgG_4_ may confer protection, as it may competes with specific IgE for allergen binding (39). However, protective mechanisms which are induced by very high exposure in the immunotherapy context may not the same to those that control baseline allergic reactivity among sensitized subjects at the population level, where exposures are at a log-scale lower range (35, 36).

### Blood Eosinophils: A Biomarker for Stratification of Asthmatic Patients

The presence of elevated eosinophil counts in blood or sputum is of considerable importance, as these cells are central effectors at the site of allergic inflammation (40). Understanding of the contribution of eosinophils in inflammatory process in asthma has attracted much research attention, leading to the development of successful therapeutic interventions. Mepolizumab is an antibody targeting interleukin (IL)-5 (41, 42), and therapeutic agents targeting IL-4 and IL-13 also show considerable promise, with multiple clinical trials over the last few years (43). However, despite increasing interest in the potential of biologics for the treatment of asthma, much of the clinical practice today still revolves around the use of inhaled corticosteroids (ICS) or leukotriene receptor antagonists (LRTA) as anti-inflammatory agents, and β_2_-agonists for short-term symptom relief. Strikingly, a study conducted in the early 1950s by the UK's Medical Research Council found that treatment of patients with chronic asthma with systemic corticosteroids showed no significant advantage over placebo (44). The fundamental problem related to this study was addressed a few years later: the lack of stratification. In one of the most important studies in asthma, Harry Morrow-Brown reported that corticosteroids are very effective, but only among patients who had a large number of eosinophils in their sputum, while the same treatment may be contra-indicated in patients without sputum eosinophilia (45). This was one of the first examples in asthma that a biomarker can be used for patient stratification to predict treatment response. More than six decades later, sputum and/or blood eosinophils are still considered the most reliable predictors of the response to corticosteroids (46). Sputum eosinophils also predict severe exacerbations, and a large study that analyzed data from ~130,000 patients found that eosinophil levels in blood were equally predictive (47). Despite this, simple investigations such as the assessment of the level of eosinophils in full blood count are rarely used in clinical practice to select patients for corticosteroid prescription, or for the assessment of future risk of exacerbations.

### Biomarkers for the Prediction of Treatment Response in Preschool Children With Asthma

A recent study which investigated the relationship between phenotypic features and biomarkers in relation to response profiles to asthma medication in young children between the ages of 12 and 59 months on the step two of asthma management identified a subgroup of patients who benefitted from the daily use of ICS. The features that predicted preferential response to regular ICS were aeroallergen sensitization and blood eosinophil counts of 300/μL (48). This brings together allergic sensitization and elevated blood eosinophils as core biomarkers of treatment response to ICS in young children, and we would argue that every pre-school child who is considered for long-term treatment with ICS should have these two features measured before the decision is made on the commencement of long-term preventive treatment. However, these two markers do not capture the full complexity of the disease. Roughly half of asthma cases do not present with eosinophilic airway inflammation, and neutrophilic inflammation may play an important role in these cases (49). These mechanisms are not as well-understood as those involved in type 2 inflammation (which is often characterized by high sIgE antibodies and eosinophil count), and many overviews point to the lack of targeted therapies for patients without type 2 inflammation (50, 51).

## Modeling Approaches to Identify Sensitization Patterns and Their Association With Asthma

### Birth Cohorts and Modeling Allergic Sensitization Patterns

To better understand the heterogeneity of childhood asthma and allergic sensitization, researchers are increasingly turning toward birth cohorts (14, 52, 53). Birth cohorts allow longitudinal collection of symptoms (such as wheezing or cough) which can be captured by questionnaires, and objective measurements including response to allergens and lung function can be recorded contemporaneously. Early studies provided a simple descriptive overview of patterns that were observed, linking allergic sensitization, and wheezing (54). To increase statistical power, consortia of birth cohorts were formed [for example, the UK Study Team for Early Life Asthma Research (STELAR) consortium combined data from five birth cohorts, enabling joint analyses for >14,000 children (55, 56)]. Making sense of the large amounts of data in birth cohorts increasingly requires the extensive use of statistical and machine learning methods that can be used to either predict and classify (supervised learning), or find patterns in the data, facilitating the identification of groups with similar characteristics known as clusters (unsupervised learning) (57).

Early attempts for phenotype classification were investigator-led, and ascribed labels based on observing simple patterns (58). A common tool used for prediction was regression analysis, which models how a particular outcome depends on other relevant variables, and this has been instrumental to establish the association between asthma and early-life sensitization, as well as the aforementioned specific role of early sensitization to mite allergens as a strong risk factor (59). In recent years, the analyses have moved to a more data-driven approach, using various types of unsupervised learning to find objective groupings [reviewed in (14, 52, 53, 60)]. Amongst most commonly used methods is latent class analysis (LCA), which introduces unobserved (latent) variables that represent group membership. These latent classes (often erroneously referred to as “phenotypes”) can then be related to observable variables such as the presence and/or severity of asthma (56, 61). An in-depth review of this method has been recently published, and lists birth cohorts that have used this approach as well as the variables that informed the discovery of classes (62). However, it is of note that a recent study has identified a number of factors, including sample size, frequency, and timing of data collection, which have a major influence on the number and type of classes identified using LCA, and thus on study conclusions (61).

Other machine learning approaches can allow us to better represent the underlying structure of the data. For example, we can use Hidden Markov Models (HMMs) as graphical models: a simple picture showing the relationships between different variables at different ages can be turned into a probabilistic clustering method (63). Early applications of this approach showed a clustering of atopic children in a birth cohort into four sensitization clusters, and a strong association with asthma could only be ascertained for children in the “multiple early sensitization” group that was phenotypically characterized by sensitivity to a wide variety of allergens from early childhood (64, 65). However, it should be cautioned that clusters identified in this way still display a certain amount of heterogeneity and may not reflect true endotypes (52). A more refined approach to their identification is needed, with a greater focus on underlying mechanisms (12).

### Component Resolved Diagnostics in Asthma Diagnosis and Prognosis

Traditionally, whole allergen extracts have been used to diagnose allergic sensitization (66). However, we can now describe sensitization in much greater detail, using component-resolved diagnostics (CRD) that measures sIgE response to a large number of allergenic molecules, or allergen components (67). This can be used to help refine the relationship between sensitization and clinical outcomes (68). Analysis which used machine learning identified three patterns of IgE responses to 112 allergenic molecules measured by a commercial CRD array, with a strong association between asthma and sensitization to a group of 27 components of plant, animal, and fungal origin (69).

Further studies investigated temporal changes of component-specific IgE responses (62, 70, 71). Using a 2-stage latent class analysis to describe longitudinal trajectories of IgE responses to eight timothy grass and seven dust mite allergenic proteins, we have shown that these patterns differed between grass and mite allergens (62). Temporal features (early vs. late onset of sensitization) were dominant in grass, and diverging patterns between group 1 and group 2 allergens in dust mite (62). Importantly, we demonstrated a clear association of different longitudinal trajectories of component-specific IgE responses with clinical outcomes; an early-onset grass trajectory and a more complex mite sensitization pattern (IgE to both group 1 and group 2 allergens) increased the risk of asthma substantially (62). In a follow-up study that looked at the whole panel of 112 allergens longitudinally, a grass/cat cluster (comprising of sIgE to grass allergen Phl p 1 and cat allergen Fel d 1) at age 5 years was a strong predictor of asthma at age 16 (71). These results suggest that it may be possible to develop interpretation algorithms for CRD multiplex arrays which practicing physicians may use to predict future risk of asthma among young sensitized children. However, we are still awaiting a clinical translation of these research observations.

A more recent approach applied network analysis to investigate interactions and connectivity patterns between component-specific IgE to 112 allergenic molecules from more than 50 sources on a CRD array and related these to the presence of asthma (72). This study has shown that in contrast to peanut allergy, in which sensitization to a specific peanut component (Ara h 2) predicts clinical reactivity (73), what predicted asthma was not IgE to any individual molecule, but the pattern of interaction between allergen component-specific IgEs (72) However, further research is required to establish whether IgE connectivity patterns can be used in diagnostic tools to facilitate asthma diagnosis in a clinical situation.

Summarizing the discussion of blood biomarkers of asthma, we would highlight the report of the expert group commissioned by the US National Institutes of Health (NIH), which recommended a multi-allergen screening to define atopy as the only core biomarker for asthma (74). In addition, total and allergen-specific IgE were described as supplemental biomarkers, along with many other dimensions of asthma, including sputum and blood eosinophils. To this we would potentially add modern CRD methods for the detailed assessment of allergic sensitization, but to incorporate these into diagnostic algorithms for asthma will require the development of appropriate interpretation tools to deliver genuine innovation for the benefits to both clinicians and patients.

## Beyond Blood Biomarkers: Other Domains of Asthma

### Clinical Guidelines and Lung Function Measures

The UK National Institute of Health and Care Excellence (NICE) proposed a diagnostic algorithm for childhood asthma in children with symptoms presenting to health-care professionals, which includes the sequential use of four measures of lung function and inflammation: spirometry (forced expiratory volume in 1 s [FEV1]/forced vital capacity [FVC] ratio), bronchodilator reversibility, fractional exhaled nitric oxide (FeNO), and peak flow variability (75). However, a study using data from the UK population-based birth cohort, collected amongst study participants with recent asthma symptoms aged 13–16 years, found a poor agreement between the proposed algorithm and asthma, questioning its clinical utility and value (76). The notion that poor lung function is associated with asthma is beyond doubt—a number of studies have shown that impaired lung function in childhood is associated with persistent wheezing (77), troublesome childhood asthma (78, 79), persistent asthma throughout the life course (80), as well as with COPD in adulthood (81). However, these observations do not translate directly into the usefulness of lung function measurement (such as spirometry) in asthma diagnosis. Several issues need to be addressed when using spirometry results as a diagnostic tool. Firstly, while the NICE guidelines propose a 70% cut-off for FEV_1_/FVC ratio, Murray et al. have shown that only very few children meet this criterion (76). It may be preferable to use an age-adjusted lower limit of normal, as this may better define airways obstruction (82).

Measurement of FeNO is considered a supplemental biomarker by the NIH due to its close tracking with eosinophilic inflammation and responsiveness to corticosteroids (75). FeNO can be used to adjust ICS dose, may be used as a marker of adherence to treatment, and may give clues about the underlying pathophysiology (83). Exhaled breath condensate has also been proposed as potentially useful biomarker, but its value for clinical practice remains unclear (84). Ascertaining the patterns of immune responses to viruses as a predictor of troublesome asthma (79) or using serum CC16 as a biomarker of diminished lung function (85) may be of value, but it remains unclear whether these can be translated into clinically useful tests.

### Genetic and Environmental Factors

A further opportunity to identify biomarkers for childhood asthma may lie in discovering genetic associates, and consistent association of asthma with variants on chromosome 17q21 has been reported (86). However, due in part to the heterogeneity of the disease, the contribution of candidate genes identified in genome-wide association studies is often small, and many different pathways have been implicated (87). Some (if not most) of these variants may impact disease susceptibility only under certain environmental conditions (i.e., are context-dependent) (88, 89).

Many environmental factors play a role in the disease trajectory (90). For example, tobacco smoke exposure increases the risk of childhood wheezing and asthma (91), and is associated with frequent asthma exacerbations (92). However, the impact of these exposures differs among individuals with different genetic predisposition (93, 94). In addition to gene-environment interactions (95), a further level of complexity stems from gene-gene interactions (96), and gene-environment correlations (97).

Overall, an elaborate multitude of interactions points to the necessity of an integrated approach to facilitate the move toward a more personalized approach to allergy and asthma (12).

## Integration of Disease Domains and Methodologies: Toward a More Complete Understanding of Asthma Pathophysiology

Given the multitude of potentially relevant biomarkers discussed thus far, it seems apparent that viewing any of them in isolation does not give us sufficient information to move toward patient stratification. While the identification of phenotypes in birth cohorts using data-driven approaches gives us an insight into the structure at the population level, it has been shown that associations with risk factors can be inconsistent across studies (60), and that there is a considerable heterogeneity within supposedly homogenous phenotypes (61). Given these observations, it is perhaps not surprising that a study which aimed to determine whether clusters of childhood asthma identified using data-driven techniques among severe asthmatic children predicted responses to treatment in several randomized controlled trials has shown that therapeutic responses were similar across the clusters (98).

**Figure 1:**
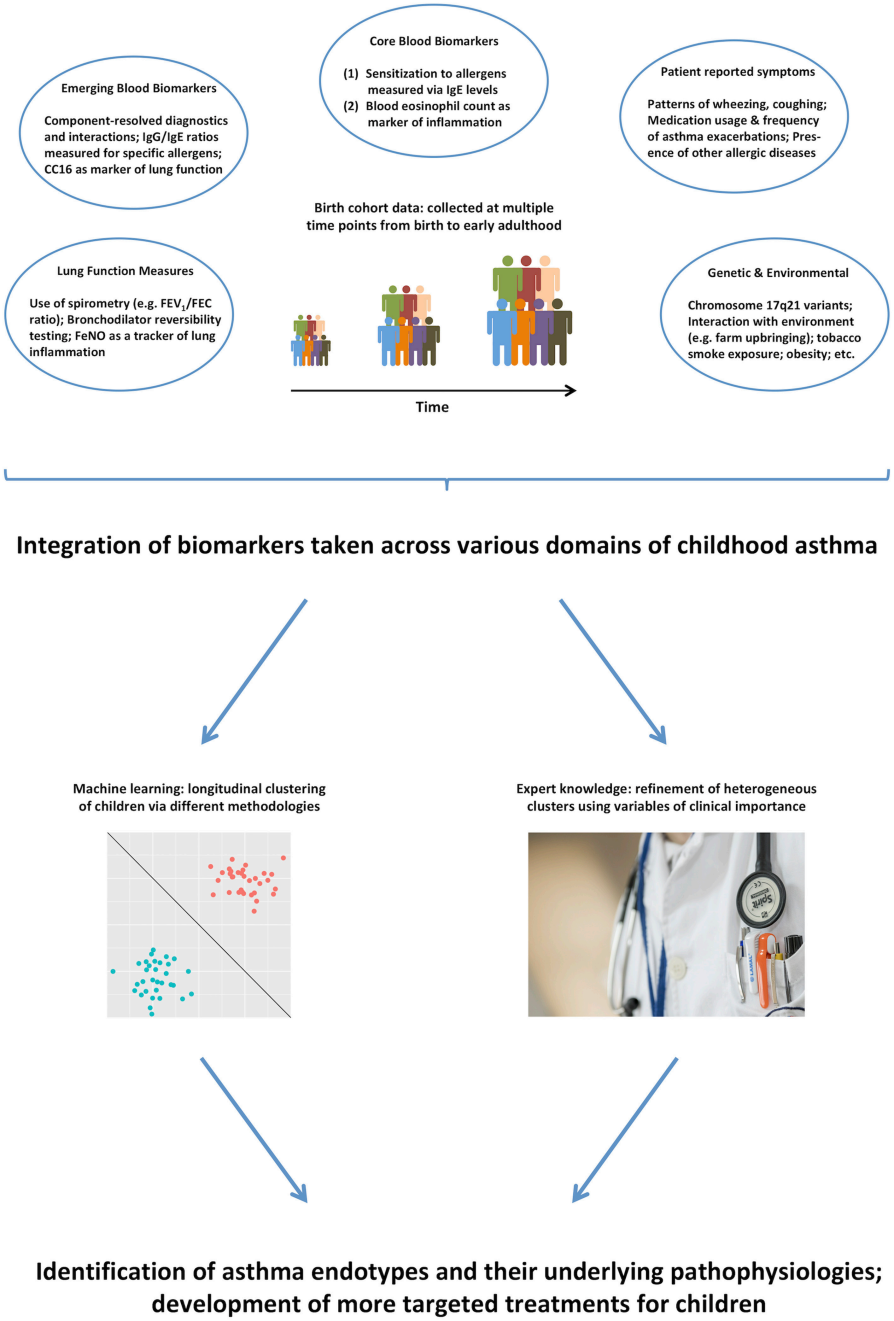
A range of different biomarkers can help us disaggregate the disease via domain integration and combining data-driven models with expert clinical knowledge.

Thus, it remains unlikely that purely data-driven identification of clusters on a subset of symptoms can identify true endotypes of this complex disease. We propose that integrating across a more comprehensive range of the available data (99), and using expert knowledge to further inform the models (100), will make results more clinically relevant, and will facilitate better understanding of the pathophysiological mechanisms underpinning different disease clusters (101). Figure 1 summarizes the key biomarkers discussed in previous sections, and illustrates how they can be used in the identification of asthma endotypes. Another complementary way to approach the identification of disease mechanism is akin to reverse engineering: stratification based on the response to particular drugs, e.g., biologics such as the aforementioned monoclonal antibodies. This is similar to the *ex juvantibus* approach sometimes used by clinicians whereby the response to treatment leads to inference about potential underlying mechanisms.

In conclusion, we urgently need better biomarkers to determine different subtypes of childhood asthma, and to predict the response to treatments. Aeroallergen sensitization and blood eosinophil count ≥300/μL may identify young pre-school children who benefit from the daily use of inhaled corticosteroids, and we suggest that every pre-school child who is considered for a long-term preventative asthma treatment should have these features measured as a minimum before the decision is made on which drug to prescribe. Several other blood biomarkers may prove useful (e.g., allergen-specific IgG/IgE antibody ratios amongst sensitized individuals, component-resolved diagnostics which measures sIgE response to a large number of allergenic molecules, level of serum CC16, etc.), but it remains unclear whether these can be translated into clinically useful tests.

## Author Contributions

All authors listed have made a substantial, direct and intellectual contribution to the work, and approved it for publication.

### Conflict of Interest Statement

AC reports personal fees from Novartis, personal fees from Regeneron/Sanofi, personal fees from Thermo Fisher Scientific, personal fees from Boehringer Ingelheim, personal fees from Novartis, personal fees from Philips, outside the submitted work. The remaining authors declare that the research was conducted in the absence of any commercial or financial relationships that could be construed as a potential conflict of interest.
